# Evaluation of a Guided Chatbot Intervention for Young People in Jordan: Feasibility Randomized Controlled Trial

**DOI:** 10.2196/63515

**Published:** 2025-02-05

**Authors:** Anne Marijn de Graaff, Rand Habashneh, Sarah Fanatseh, Dharani Keyan, Aemal Akhtar, Adnan Abualhaija, Muhannad Faroun, Ibrahim Said Aqel, Latefa Dardas, Chiara Servili, Mark van Ommeren, Richard Bryant, Kenneth Carswell

**Affiliations:** 1 Department of Mental Health, Brain Health and Substance Use World Health Organization Geneva Switzerland; 2 Institute for Family Health King Hussein Foundation Amman Jordan; 3 School of Psychology University of New South Wales Sydney Australia; 4 Department of Clinical Neuroscience Division of Insurance Medicine Karolinska Institutet Stockholm Sweden; 5 Community Health Nursing Department School of Nursing University of Jordan Amman Jordan

**Keywords:** chatbot, youth, depression, anxiety, feasibility study, randomized controlled trial, mental health, evaluation, Jordan, CBT, psychological treatment, digital intervention, health intervention, mood disorder, digital health

## Abstract

**Background:**

Depression and anxiety are a leading cause of disability worldwide and often start during adolescence and young adulthood. The majority of young people live in low- and middle-income countries where there is a lack of mental health services. The World Health Organization (WHO) developed a guided, nonartificial intelligence chatbot intervention called Scalable Technology for Adolescents and youth to Reduce Stress (STARS) to reduce symptoms of depression and anxiety among young people affected by adversity.

**Objective:**

The objective of this study was to evaluate the feasibility of the STARS intervention and study procedures among young people in Jordan.

**Methods:**

A 2-arm, single-blind, feasibility randomized controlled trial was conducted among 60 young people aged 18 years to 21 years living in Jordan with self-reported elevated levels of psychological distress. Immediately after baseline, participants were randomized 1:1 into the STARS intervention or enhanced care as usual (ECAU). STARS consisted of 10 lessons in which participants interacted with a chatbot and learned several cognitive behavioral therapy strategies, with optional guidance by a trained e-helper through 5 weekly phone calls. ECAU consisted of a static web page providing basic psychoeducation. Online questionnaires were administered at baseline (week 0) and postassessment (week 8) to assess depression (Hopkins Symptom Checklist-25 [HSCL-25]), anxiety (HSCL-25), functional impairment (WHO Disability Assessment Schedule [WHODAS] 2.0), psychological well-being (WHO-Five Well-Being Index [WHO-5]), and agency (State Hope Scale). Process evaluation interviews with stakeholders were conducted after the postassessment.

**Results:**

Participants were recruited in December 2022 and January 2023. Of 700 screening website visits, 160 participants were eligible, and 60 participants (mean age 19.7, SD 1.16 years; 49/60, 82% female) continued to baseline and were randomized into STARS (n=30) or ECAU (n=30). Of those who received STARS, 37% (11/30) completed at least 8 chatbot lessons, and 13% (4/30) completed all 5 support calls. The research protocol functioned well in terms of balanced randomization, high retention at postassessment (48/60, 80%), and good psychometric properties of the online questionnaires. Process evaluation interviews with STARS participants, ECAU participants, e-helpers, and the clinical supervisor indicated the acceptability of the study procedures and the STARS and ECAU conditions and highlighted several aspects that could be improved, including the e-helper support and features of the STARS chatbot.

**Conclusions:**

This study demonstrated the feasibility and acceptability of the STARS intervention and research procedures. A fully powered, definitive randomized controlled trial will be conducted to evaluate the effectiveness of STARS.

**Trial Registration:**

ISRCTN ISRCTN19217696; https://doi.org/10.1186/ISRCTN19217696

## Introduction

Globally, 1 in 8 people live with a mental disorder, and among adolescents, the rate is 1 in 7 [1]. Anxiety and depression are the most common mental disorders, affecting 5.5% and 3.5%, respectively, of young people aged 15 years to 19 years globally in 2021 [1]. The majority of young people live in low- and middle-income countries (LMIC) where services, especially for young people, are often scarce due to a lack of government policy on mental health, funding, and trained mental health professionals [2]. Additionally, there is a paucity of research on psychological interventions for young people in LMIC [3-5].

Digital mental health or e–mental health shows promise as a strategy for reducing symptoms of common mental health conditions. A recent meta-analysis showed that, among adults and young people, mental health smartphone apps have small but significant effects on reducing depression and anxiety, with larger effect sizes when cognitive behavioral therapy strategies, chatbot technology (ie, a computer simulating a human conversation), or mood monitoring are included [6]. In addition, digital interventions for young people (12-25 years) were found to be mostly effective when guided (in which a client is provided motivation and support by a therapist or researcher) [7]. Among the advantages of digital interventions are reduced reliance on human resources, greater access and availability, and the potential to overcome concerns about stigmatization. Among the challenges are poor engagement and adherence [8].

Research on chatbots that provide psychological interventions has grown significantly. The majority of chatbots included in a 2019 review investigated rule-based chatbots (ie, depending on decision trees), whereas few investigated artificial intelligence (AI)–based chatbots (ie, reliant on machine learning). Although AI-based chatbots better mimic natural conversations, can apply therapeutic techniques dynamically, and learn over time, they are a new technology, and possible risks and challenges with large language models are currently unclear [9]. Rule-based chatbots operate with predefined scripts and pathways and are therefore predictable and allow for standardization of interventions. The World Health Organization (WHO) has developed, tested, and released low-intensity or potentially scalable psychological interventions [10,11]. This includes digital interventions to reach a larger proportion of the population, due in part to the majority of the global population (66%) now having access to the internet [12]. Previous studies have confirmed the effectiveness of a WHO digital intervention that uses behavioral activation for adults, including Syrian refugees, with depression [13,14]. Building on this promising work, WHO developed a digital intervention specifically aimed at young people called Scalable Technology for Adolescents and youth to Reduce Stress (STARS), which uses a rule-based chatbot interface to increase engagement and teach a range of psychological techniques [15]. The intervention was developed using a human-centered design approach in which 269 youth and 86 community members from Pakistan, Jamaica, occupied Palestinian territories, South Africa, and Nepal and 20 clinical psychology experts were involved [15]. Because, at the time of testing and development, AI-powered chatbots were a very new technology, a decision was made to use a rule-based chatbot to ensure a greater level of predictability and control.

The objective of this study was to evaluate the feasibility of the STARS intervention and study procedures among young people in Jordan. WHO had initially planned to test STARS with 15-year-olds to 18-year-olds in schools in Jordan as there are few evidence-based psychological interventions available for this age group, especially in LMIC. However, COVID-19 meant that accessing schools was not feasible. To test STARS with a group as close to the intended original age range as possible, while managing the pragmatic realities of recruitment of people younger than 18 years, a decision was taken to test it instead with youth aged 18 years to 21 years.

## Methods

### Design and Setting

A 2-arm, single-blind, feasibility randomized controlled trial (RCT) including a process evaluation was conducted in Jordan, with study implementation coordinated by the Institute of Family Health (IFH). Jordan is a lower middle-income country [16] with a population of 11.5 million people [17], of which there are about 3 million Palestinians [18] and 1.3 million Syrians [19]. Young people in Jordan, especially refugees, face barriers to education [20], high unemployment rates [20,21], and increasing poverty [22].

### Ethical Considerations

The trial was approved by the research committee of the School of Nursing, University of Jordan (PF.22.9 on March 23, 2022) and the Ethics Review Committee at WHO (ERC.0003729 on July 1, 2022). The CONSORT (Consolidated Standards of Reporting Trials) guideline for feasibility studies is available in Multimedia Appendix 1 [23]. Digital informed consent was obtained from all participants before screening on the screening website. Participant data were collected online through the Qualtrics data collection software and deidentified after data collection was complete. Participants received a compensation of JD 3 (US $4.23) upon completion of the postassessment. Only members of the research team had access to the data.

### Procedures

We aimed to recruit 60 participants (30 in each condition; cf [24]) through IFH peer educators (educators and volunteers at IFH who provide health-related information to youth through various activities), universities, and online advertising on Facebook and Instagram. Participants were included if they met the following criteria: (1) aged between 18 years and 21 years; (2) lived in Jordan; (3) elevated levels of psychological distress, as indicated by a score of 20 or higher on the Kessler Psychological Distress Scale (K10) [25,26]; and (4) had access to a device for intervention delivery or were willing to use one at a participating center. Participants who met all inclusion criteria were asked to complete screening questions on the imminent risk of suicide [27]. Those who answered “yes” to having taken actions to end their life or who answered “yes” or “unsure” to having a plan to end their life in the next 2 weeks were shown a message encouraging them to seek professional care as soon as possible. The message provided referral options to mental health services in Jordan, including the phone number of the clinical supervisor at IFH. Participants at imminent risk of suicide were excluded from the trial.

Participants who screened positive were then contacted by phone by the project manager for a research engagement call in which participants were reminded of the study procedures and could ask any further questions; after the call, they received a personalized baseline link. Upon completion of the baseline link, participants were randomized by the Qualtrics questionnaire tool on a 1:1 ratio to either STARS or enhanced care as usual (ECAU). The research manager then created participants’ accounts for the STARS or ECAU website and allocated STARS participants to an e-helper (a trained and supervised nonspecialist offering additional support sessions over the phone). The intervention period lasted 8 weeks followed by the postassessment.

### Conditions

#### STARS Intervention

The STARS intervention uses a cognitive behavioral therapy framework to address symptoms of depression and anxiety in young people. This approach is consistent with WHO guidelines of psychological treatments for young people [28]. STARS is a non-AI decision tree logic-based conversational agent (chatbot) delivered through a website. The intervention consists of 10 brief lessons, each taking approximately 20 minutes to complete. The lessons are delivered in a conversational, text messaging style by a chatbot called “Salam.” The name Salam was chosen during preliminary adaptation work in Jordan as it is a gender-neutral Arabic name and greeting that means peace. During the chatbot lessons, participants are introduced to the intervention (lesson 1) and learn several self-help strategies, including psychoeducation (lesson 2), emotion regulation (lessons 3 and 4), behavioral activation (lessons 5 and 6), problem management (lesson 7), thought challenging (lessons 8 and 9), and relapse prevention (lesson 10). Participants are encouraged to practice and apply the strategies in their daily lives. A “toolbox” provides support files, such as stress management audios and short video clips, and information on other sources of mental health and psychosocial support in Jordan, including emergency help and contact details to local service providers. Participants are encouraged to complete 2 chatbot lessons a week and to complete all 10 chatbot lessons within the 8-week intervention period. Participants can continue to access the chatbot and their account during the whole study period (ie, also after their postassessment).

In addition to the chatbot, participants are offered 5 calls lasting approximately 15 minutes by an e-helper delivered over a maximum period of 8 weeks. The aim of the calls is to support participants and increase motivation and adherence to the intervention. e-Helpers received 5 days of training and weekly group supervision with a clinical psychologist. The project coordinator attended 10% of calls to rate fidelity to the e-helper manual using a fidelity checklist.

#### Enhanced Care as Usual

This condition included psychoeducation about depression and anxiety. Psychoeducation was delivered via a static website (not a chatbot) that participants could access for the whole study period after registering via a personal invitation link. The content of the psychoeducation was very similar to the content of lesson 2 of the STARS chatbot and included psychoeducation about emotions, a personal story about a fictional character who talks about her emotions, and information about where to access mental health support. It included a list of organizations providing mental health and psychosocial support in Jordan. This list was also included in the “toolbox” section of the chatbot for STARS intervention participants (see the STARS Intervention section). Participants in the ECAU condition did not receive additional e-helper support.

### Feasibility Measures

The primary outcome of this study was the feasibility of the STARS intervention and study among youth aged 18 years to 21 years in Jordan. Feasibility was assessed using the following criteria: (1) recruitment rate recorded as the number of eligible participants who consented to participate in the study, (2) dropout rates recorded as the number of randomized participants who did not complete the intervention nor the postassessment, (3) number of adverse events and serious adverse events, and (4) evaluation of (secondary) outcome measures and estimation of differences across the 2 groups. In addition, a qualitative process evaluation was conducted to understand aspects related to implementing the STARS intervention and study.

### Secondary Outcome Measures

Psychological distress was measured with the K10 [25,26]. It includes 10 questions scored on a scale from 1 to 5 (sum score range: 10-50), with higher scores indicating higher psychological distress. The measure has been validated among Palestinians with good internal consistency [25]. Symptoms of anxiety and depression in the last week were assessed with the 25-item Hopkins Symptom Checklist (HSCL-25) [29]. Questions are scored on a scale from 1 to 4, with higher scores indicating greater distress. The total scores for the subscales are the average of the 10 anxiety symptoms and the average of the 15 depression symptoms. The instrument has been validated in Arabic-speaking populations with good psychometric properties [30,31]. Functional impairment was measured with the 12-item version of the WHO Disability Assessment Schedule (WHODAS 2.0) [32], which covers 6 domains of functioning (ie, cognition, mobility, self-care, getting along, life activities, and participation) over the past 30 days. Items are scored on a scale from 0 to 4 (total sum score: 0-48), with a higher score indicating greater functional impairment. The instrument has been widely used and across various populations [33] and performed well in terms of psychometric properties among Syrian refugees [34]. The sociodemographic section of the WHODAS 2.0 was adapted to collect data on age, gender, nationality, living situation, education level, marital status, and work status. Self-identified problems were assessed with the Psychological Outcomes Profiles (PSYCHLOPS) instrument [35]. Items are rated on a scale from 0 to 5 (total sum score 0-20) referring to the last week. The instrument was previously used in Arabic-speaking populations [36]. Psychological well-being was measured with the WHO-Five Well-Being Index (WHO-5) [37], a widely used instrument in which 5 positively framed statements about well-being in the past 2 weeks are rated on a scale from 0 to 5, with higher scores indicating better well-being. The Arabic translation has been validated in a Saudi sample [38]. Agency was assessed through the agency subscale of the State Hope Scale [39], which consists of 3 items of perceived levels of goal-directed energy measured on a scale from 1 to 8. Higher scores indicate higher levels of perceived agency.

Additionally, satisfaction with the intervention was assessed with the Client Satisfaction Questionnaire for internet-based interventions (CSQ-I) [40] at the postassessment only. All secondary outcome measures are presented in Table 1.

**Table 1:** Secondary outcome measures.

Domain	Instrument	Time frame	Number of items	Screening (T0)	Baseline (T1)	Intervention	Postassessment (T2)
Psychological distress	K10^a^	30 days	10	X	—^b^	—	X
Depression symptoms	HSCL-25^c^	7 days	15	—	X	—	X
Depression symptoms	PHQ-2^d^	14 days	2	—	—	X	—
Anxiety symptoms	HSCL-25	7 days	10	—	X	—	X
Functional impairment	WHODAS^e^ 2.0	30 days	12	—	X	—	X
Subjective well-being	WHO-5^f^	14 days	5	—	X	—	X
Self-identified problems	PSYCHLOPS^g^	Currently	7 (T1) or 8 (T2)	—	X	—	X
Agency	SHS-A^h^	Currently	3	—	X	—	X
User satisfaction	CSQ-I^i^	Currently	8	—	—	—	X

^a^K10: Kessler Psychological Distress Scale.

^b^Not applicable.

^c^HSCL-25: Hopkins Symptom Checklist-25.

^d^PHQ-2: Patient Health Questionnaire.

^e^WHODAS: World Health Organization Disability Assessment Schedule.

^f^WHO-5: World Health Organization-Five Well-being Index.

^g^PSYCHLOPS: Psychological Outcomes Profiles.

^h^SHS-A: State Hope Scale agency subscale.

^i^CSQ-I: Client Satisfaction Questionnaire for internet-based interventions.

### Quantitative and Qualitative Analyses

Descriptive statistics are presented using mean values, SDs, frequencies (n), and percentages. Cronbach α was used to evaluate the internal reliability of study instruments at baseline, and correlation analysis was used to evaluate convergent validity across instruments. Baseline differences were tested using chi-square tests for frequencies and independent-sample *t* tests for continuous variables.

As this was a feasibility RCT aimed to evaluate the feasibility of the STARS intervention, it was not powered to detect significant differences. However, to evaluate the secondary outcome measures and estimate differences across the 2 groups, univariate completers analyses of covariance (ANCOVA) for all outcome measures were performed, with their respective baseline scores as covariates. Only participants who completed the postassessment were included in the statistical analyses. Prorated imputation was used for participants with maximally 20% missing values on the scale (ie, using the participant’s mean score of the other items of the scale). Total scores were considered missing for participants if more than 20% of items were missing on the scale.

Subsequent to the trial, a qualitative process evaluation was conducted with 5 key informant groups: (1) STARS completers, (2) STARS noncompleters, (3) ECAU participants, (4) e-helpers, and (5) the clinical supervisor. The number of key informants per group was predefined in the study protocol based on comparable studies (eg, [41,42]) and feasibility given the low number of participants and staff in this study. We followed the following procedure to select key informants for an interview. The local research coordinator first categorized participants as being a STARS completer (ie, having completed at least 8 chatbot lessons), STARS noncompleter (ie, having completed less than 8 chatbot lessons), or ECAU participant. The research coordinator then contacted participants from each group in order of enrollment date and stopped inviting new participants when a sufficient number of key informants per group was reached (ie, 4 to 5 key informants per group). Given the small helper team during the feasibility study, all helpers (n=2) and the only clinical supervisor were invited to participate in the process evaluation. Key informants all provided informed consent to participate in the process evaluation interview including being audio recorded and did not receive (additional) compensation for their participation. The topics covered in the semistructured interviews are outlined in Table 2. Interviews were conducted over the phone (STARS and ECAU participants) or face-to-face (e-helpers and supervisor) and audio recorded for transcription. Transcripts were transcribed verbatim to the language of the interview and deidentified prior to analysis. Deidentified Arabic transcripts were independently analyzed by 2 Arabic-speaking researchers (RH and SF) using thematic analysis. Categorization of responses was checked between the researchers. Key informant groups were analyzed separately, and data were compared to identify commonalities and differences in responses. Results were discussed with a third researcher (AMDG).

**Table 2:** Process evaluation interview topics.

Key informant group	Interview topics
STARS^a^ completers and noncompleters	Overall impressions of the STARS intervention and study Reason for discontinuation (if applicable) Views on the STARS intervention Rapport with the e-helper Research process
ECAU^b^ participants	Overall impressions of the ECAU condition and study Views on the ECAU condition Research process
e-Helpers	Overall impressions of the STARS intervention and study Views on the STARS intervention Rapport with participants e-Helper support calls Local management and supervision
Clinical supervisor	Overall impressions of the STARS intervention and study Integrating the role of supervisor in the workload Training, supervision, and retention of e-helpers

^a^STARS: Scalable Technology for Adolescents and youth to Reduce Stress.

^b^ECAU: enhanced care as usual.

## Results

### Feasibility of STARS

#### Recruitment

**Figure 1:**
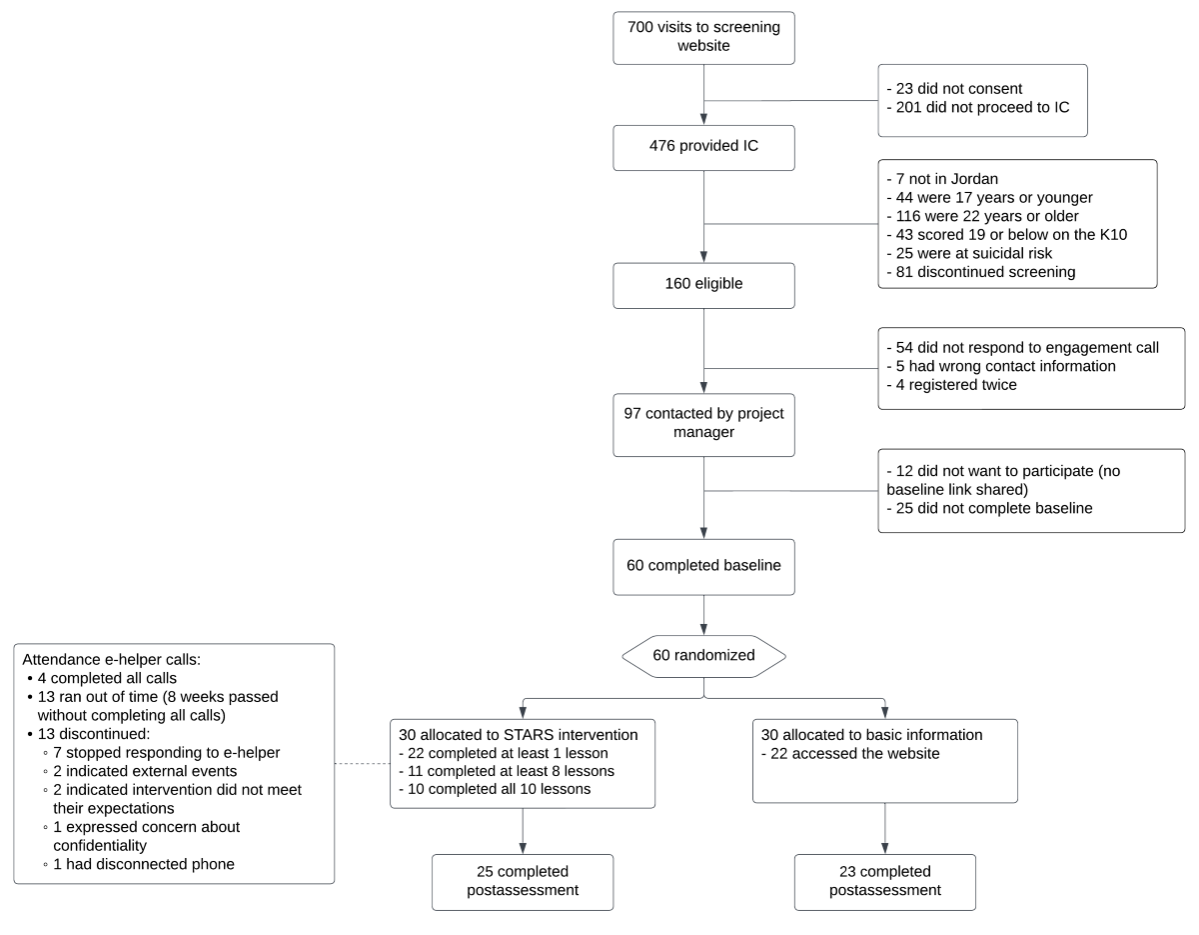
CONSORT (Consolidated Standards of Reporting Trials) flow diagram. IC: informed consent; K10: Kessler Psychological Distress Scale; STARS: Scalable Technology for Adolescents and youth to Reduce Stress.

Participants were recruited through IFH peer educators, universities in Amman, and online advertising between December 1, 2022, and January 26, 2023. Of 700 visits to the screening website, 476 provided informed consent and completed the screening questionnaires. Of those, 160 participants were eligible to participate and called by the project manager for an “engagement call.” Of those, 54 did not respond to the engagement call, 25 did not proceed to baseline, 12 indicated they did not want to participate, 5 did not provide correct contact details, and 4 registered twice (see Figure 1). The 60 participants who continued to baseline heard about the study through a friend (n=28), social media (n=13), an IFH peer educator (n=10), or from another source (n=9).

#### Group Allocation and Registration to the STARS Website

Upon completion of the baseline assessment, 60 participants were randomized into STARS (n=30) or ECAU (n=30). The project manager assigned participants to either a control facilitator or e-helper who created a condition-specific account for the participant. Of the 60 participants invited, 52 participants registered at the STARS website, of which 26 were STARS chatbot participants and 26 were ECAU participants.

#### Participant Characteristics

The baseline sample of 60 participants consisted of 49 women (82%) and 11 men (18%) between the ages of 18 years and 21 years (mean 19.7, SD 1.16 years; Table 3). Participants identified as Jordanian (31/60, 52%), Syrian (27/60, 45%), or other (2/60, 3%). The majority (53/60, 88%) were unmarried. Most participants (40/60, 67%) reported a general secondary education or a bachelor’s degree as their highest level of education started and indicated that they were currently a student (28/60, 47%). There were no statistically significant differences in baseline characteristics between the STARS and ECAU conditions.

**Table 3:** Baseline characteristics.

Characteristics			Total sample (N=60)		STARS^a^ intervention (n=30)		Control condition (n=30)		Comparison statistic (*df*)			*P* value	
Gender (male), n (%)			11 (18)		5 (17)		7 (23)		0.11 (1)^b^			.73	
Age (years), mean (SD)			19.68 (1.16)		19.43 (1.10)		19.93 (1.27)		1.70 (58)^c^			.94	
Age (years), range			18-21		18-21		18-21		—^d^			—	
**Nationality, n (%)**										3.22 (2)^b^			.20
	Jordanian	31 (52)		17 (57)		14 (47)							
	Syrian	27 (45)		11 (37)		16 (53)							
	Other (Palestinian, Iraqi)	2 (3)		2 (7)		0 (0)							
**Highest education started, n (%)**										6.94 (5)^b^			.22
	No education	2 (3)		2 (7)		0 (0)							
	Basic education	9 (15)		5 (17)		4 (13)							
	Technical/vocational secondary education	5 (8)		1 (3)		4 (13)							
	Technical diploma	4 (7)		3 (10)		1 (3)							
	General secondary education	15 (25)		5 (17)		10 (33)							
	Bachelor’s degree	25 (42)		14 (47)		11 (37)							
**Marital status, n (%)**										2.19 (3)^b^			.52
	Never married	53 (88)		27 (90)		26 (87)							
	Currently married	5 (8)		2 (7)		3 (10)							
	Separated	1 (2)		0 (0)		1 (3)							
	Divorced	1 (2)		1 (3)		0 (0)							
**Work status, n (%)**										7.39 (6)^b^			.28
	Paid work	11 (18)		3 (10)		8 (27)							
	Nonpaid work	1 (2)		1 (3)		0 (0)							
	Self-employed	0 (0)		0 (0)		0 (0)							
	Student	28 (47)		14 (47)		14 (47)							
	Keeping house	8 (13)		5 (17)		3 (10)							
	Retired	0 (0)		0 (0)		0 (0)							
	Unemployed (health reasons)	0 (0)		0 (0)		0 (0)							
	Unemployed (other reasons)	7 (12)		5 (17)		2 (7)							
	Other	3 (5)		2 (7)		1 (3)							
	Not allowed to work in Jordan	2 (3)		0 (0)		2 (7)							

^a^STARS: Scalable Technology for Adolescents and youth to Reduce Stress.

^b^Chi-square test.

^c^*t* test.

^d^Not applicable.

#### Adherence to STARS and ECAU Conditions

In total, 26 STARS and 22 control participants accessed the content of their intervention following allocation. For the STARS intervention, this meant accessing at least the home page, and for the control group, this meant accessing the psychoeducation page. Within the 8-week intervention period, 22 of the 30 STARS participants (73%) completed at least 1 chatbot lesson, 11 participants (37%) completed at least 8 chatbot lessons, and 10 participants (33%) completed all 10 chatbot lessons, with an average of 4.47 lessons completed. Table 4 shows the number of participants completing each lesson.

**Table 4:** Scalable Technology for Adolescents and youth to Reduce Stress (STARS) intervention attendance of chatbot lessons and e-helper support calls (n=30).

Lesson or call number		Participants who completed, n (%)	Duration of call (minutes), mean (SD)
**Lesson**			
	1	22 (73)	—^a^
	2	17 (57)	—
	3	14 (47)	—
	4	14 (47)	—
	5	12 (40)	—
	6	12 (40)	—
	7	11 (37)	—
	8	11 (37)	—
	9	11 (37)	—
	10	10 (33)	—
**e-Helper call**			
	1	21 (70)	7.3 (2.1)
	2	13 (43)	8.3 (2.6)
	3	11 (37)	7.5 (1.9)
	4	9 (30)	8.4 (3.2)
	5	4 (13)	7.3 (3.0)

^a^Not applicable.

In terms of the e-helper support calls, 21 of the 30 participants (70%) attended at least 1 support call, and 4 of the 30 participants (13%) attended all 5 support calls. The average number of calls attended was 1.93, and the average call duration was 7.8 (SD 2.4; range 4-14) minutes. Of the 26 participants who did not attend all support calls, 13 participants ran out of time (ie, 8 weeks had passed without completing all calls), and 13 participants did not continue attending the support calls. In addition, 7 participants stopped responding to the e-helper (ie, as per the standard operating procedure, the e-helper stopped contacting the participant after 3 consecutive unsuccessful attempts to get in touch), 2 participants indicated external events hindered them from continuing the intervention (ie, passing of family member, exams), 2 participants expressed the intervention did not meet their expectations (eg, looking for a job), 1 participant stopped after expressing concern about confidentiality of the chatbot, and 1 participant’s phone was disconnected. Table 4 presents the attendance at the STARS chatbot lessons and e-helper support calls.

#### STARS Protocol Fidelity Assessment of e-Helper Phone Calls

A total of 15 e-helper phone calls were attended and assessed on fidelity (call 1: n=4; call 2: n=3; call 3: n=4; call 4: n=2; call 5: n=2). Fidelity assessments indicated that 98% of the call content was carried out.

#### Serious Adverse Events

No adverse events nor serious adverse events were reported.

#### Retention at Postassessment

The postassessment, at 8 weeks after baseline, between January 26, 2023, and March 27, 2023, was completed by 48 (80%) of the 60 participants, of which 25 (25/30, 83%) were STARS intervention participants and 23 (23/30, 77%) were control group participants. Reasons for dropout at postassessment were not provided due to the online nature of the study.

### Secondary Outcome Measures

All outcome measures had good internal consistency with a Cronbach α >0.80 (see Table 5). Across assessments completed by participants, 2.7% (180/6576) of the outcome questionnaire items were missing.

**Table 5:** Baseline comparison of scores on secondary outcome measures and internal consistency.

Domain	Measure	Possible range	Validated cutoff	Total (N=60), mean (SD; range)	STARS^a^ (n=30), mean (SD)	ECAU^b^ (n=30), mean (SD)	Comparison			Cronbach α
							Statistic (*df*)	*P* value		
Suicidal thoughts past month	Single item	Yes/no	—^c^	10 (17)^d^	3 (10)^d^	7 (23)^d^	1.92 (1)^e^	.29	—	
Psychological distress	K10^f^	10-50	≥20^g^	32.77 (7.31; 20-48)	31.53 (7.82; 20-48)	34.00 (6.66; 21-46)	–1.32 (58)^h^	.19	0.84	
Functional impairment	WHODAS^i^	0-48	≥17^j^	26.68 (8.66; 14-50)	26.73 (10.11; 14-50)	26.63 (7.34; 14-43)	–0.07 (55)^h^	.94	0.88	
Anxiety and depression combined	HSCL-25^k^	1-4	—	2.46 (0.69; 1.04-3.70)	2.39 (0.73; 1.04-3.60)	2.54 (0.65; 1.20-3.70)	–0.86 (57)^h^	.19	0.94	
Anxiety	HSCL-25 subscale	1-4	≥2.00^l^	2.38 (0.70; 1.00-3.60)	2.60 (0.67; 1.27-3.93)	2.46 (0.68; 1.00-3.50)	–0.89 (56)^h^	.37	0.88	
Depression	HSCL-25 subscale	1-4	≥2.10^m^	2.52 (0.72; 1.07-3.93)	2.45 (0.76; 1.07-3.67)	2.30 (0.71; 1.00-3.60)	–0.83 (57)^h^	.41	0.91	
Self-identified problems	PSYCHLOPS^n^	0-20	—	13.66 (5.49; 0-20)	13.54 (4.96; 0-20)	13.89 (6.14; 0-20)	–0.17 (51)^h^	.86	0.90	
Psychological well-being	WHO-5^o^	0-25	<50^p^	36.93 (22.29; 0-100)	42.27 (24.38; 4-100)	31.60 (18.91; 0-84)	1.89 (58)^h^	.06	0.87	
Agency	SHS^q^ agency	3-24	—	12.32 (5.29; 3-23)	12.73 (5.29; 3-23)	11.90 (5.34; 3-21)	0.61 (58)^h^	.54	0.87	

^a^STARS: Scalable Technology for Adolescents and youth to Reduce Stress.

^b^ECAU: enhanced care as usual.

^c^Not applicable.

^d^n (%).

^e^Chi-square test.

^f^K10: Kessler Psychological Distress Scale.

^g^Cutoff for probable anxiety or affective disorder with a sensitivity of 0.66 and specificity of 0.92 [43].

^h^*t* test.

^i^WHODAS: World Health Organization Disability Assessment Schedule.

^j^Indicator of moderate impairment [34].

^k^HSCL-25: Hopkins Symptom Checklist-25.

^l^Cutoff for probable anxiety disorder with a sensitivity of 0.84 and a specificity of 0.59 [31].

^m^Cutoff for probable depressive disorder with a sensitivity of 0.82 and a specificity of 0.70 [31].

^n^PSYCHLOPS: Psychological Outcomes Profiles.

^o^WHO-5: World Health Organization-Five Well-Being Index.

^p^Cutoff for depression with a sensitivity of 0.86 and a specificity of 0.81 [37].

^q^SHS: State Hope Scale.

**Figure 2:**
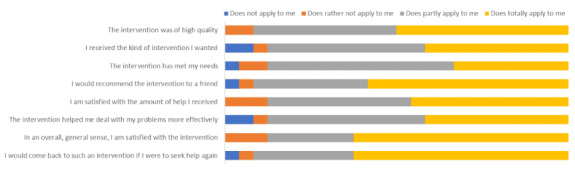
Results of the Client Satisfaction Questionnaire for internet-based interventions (CSQ-I; n=24).

Table 6 presents the baseline mean values for the HSCL-25 (total score and anxiety and depression subscales), K10, WHODAS 2.0, PSYCHLOPS, WHO-5, and State Hope Scale agency subscale indicating no significant differences between groups at baseline. The ANCOVA of postassessment scores on the HSCL-25 controlling for baseline HSCL-25 scores indicated a nonsignificant effect of condition (STARS: mean 2.26, SD 0.79; ECAU: mean 2.52, SD 0.51; *F*_1, 43_=0.42, *P*=.52, ƞ=0.01). Univariate ANCOVAs also indicated nonsignificant effects of condition on all other outcomes (see Table 6). The mean score on the CSQ-I to evaluate satisfaction with the intervention was 26.79 (SD 5.07; n=24). Figure 2 summarizes the responses to each statement on the CSQ-I.

**Table 6:** Univariate completer analysis of covariance (ANCOVA) results for each outcome measure.

Outcome	Postassessment scores, mean (SD)			ANCOVA		
	STARS^a^	ECAU^b^	*F* (*df*)		*P* value	Effect size, ƞ
HSCL-25^c^ total	2.26 (0.79)	2.52 (0.51)	0.42 (1,43)		.52	0.01
HSCL-25 anxiety	2.21 (0.78)	2.39 (0.61)	0.02 (1,42)		.88	0.00
HSCL-25 depression	2.29 (0.84)	2.62 (0.52)	0.91 (1,43)		.34	0.02
WHODAS^d^ 2.0	25.39 (8.67)	26.77 (6.82)	0.45 (1,42)		.50	0.01
K10^e^	28.20 (9.17)	30.00 (8.63)	0.02 (1,43)		.90	0.00
PSYCHLOPS^f^	10.96 (5.46)	13.30 (4.40)	3.21 (1,41)		.08	0.07
WHO-5^g^	49.12 (23.99)	35.27 (20.27)	2.18 (1,44)		.14	0.05
SHS^h^ agency	14.58 (4.37)	13.86 (5.20)	0.01 (1,43)		.90	0.00

^a^STARS: Scalable Technology for Adolescents and youth to Reduce Stress.

^b^ECAU: enhanced care as usual.

^c^HSCL-25: Hopkins Symptom Checklist-25.

^d^WHODAS: World Health Organization Disability Assessment Schedule.

^e^K10: Kessler Psychological Distress Scale.

^f^PSYCHLOPS: Psychological Outcomes Profiles.

^g^WHO-5: World Health Organization-Five Well-Being Index.

^h^SHS: State Hope Scale.

### Process Evaluation

Semistructured interviews were conducted between February 14, 2023, and March 19, 2023, with 4 STARS completers, 5 STARS noncompleters, 4 ECAU participants, 2 e-helpers, and 1 clinical supervisor (n=16). Themes included perspectives on the study procedures, STARS intervention, e-helper support, technical aspects of the chatbot, and ECAU condition.

### Study Procedures

Most participants found completing the questionnaires “easy” but lengthy and suggested clearer explanations of the randomization process. Regarding the assessment of protocol fidelity, 1 e-helper expressed discomfort with live ratings and suggested the use of audio recordings of calls instead.

### General Views of the STARS Intervention

Generally, participants held positive views regarding the STARS chatbot, found the lessons informative (eg, the skills learned), enjoyed the human-like interaction, and appreciated the use of informal Arabic to mimic text messaging among young people. The supervisor was positive about the intervention overall including its possible ability to tackle stigma yet was uncertain about the minimal level of human contact. Additional content such as more chatbot lessons or longer character “stories” was desired by 4 STARS completers. All 4 STARS completers reported using the therapeutic skills and stress management audios in their daily life. STARS noncompleters acknowledged the benefits of the skills, but they did not apply them routinely. One e-helper noticed the benefits of the skills for herself as well.

### Views on and Engagement With e-Helper Support

Views on the e-helper support were mixed. Although e-helpers were described as “friendly,” “good listeners,” and showing understanding, there were also suggestions for improvements, such as using a more caring tone. One e-helper emphasized the importance of building rapport and to encourage participants to use the chatbot. There were also other opinions expressed but no clear consensus. For example, e-helpers noted varying participant preferences for the level of contact; 1 STARS completer suggested e-helpers should be available 24/7, while 1 participant dropped out because they thought their e-helper might be able to read the chatbot conversations. e-Helpers felt that young people were hesitant to share much over the phone, and some participants preferred expressing themselves through text messages. e-Helpers expressed a need for more training to conduct the 15-minute support calls. All STARS completers said the 15-minute call duration was suitable.

### Engagement With the STARS Chatbot

Reasons for not completing the intervention included factors such as technical difficulties; concerns about the privacy of the chatbot lessons; the intervention being too “simple” to address problems; and external factors, such as feeling too stressed and having a health issue. Two participants suggested adding videos about the skills to enhance engagement, 3 participants suggested follow-up calls after the study was over (eg, a check-in), and 1 suggested a “modular” instead of sequential chatbot. Last, 1 e-helper suggested a call before participants start their chatbot lessons so that the e-helper can explain more about the intervention and support participants with registration.

### Technical Aspects of the STARS Chatbot

STARS participants mentioned that the intervention was user-friendly (eg, conversational chatbot) but suggested several improvements. More flexibility in responding to the chatbot (eg, open response formats rather than predefined response options) was preferred by 2 STARS dropouts, and 3 suggested the use of notifications as reminders to complete the lessons. One e-helper and some participants noted the problems created by using email to register, as some people will not be familiar with it. Other technical limitations included expiry of a sign-up link within 24 hours, the need to restart a chatbot lesson in case of internet disconnection, and the presence of various bugs that hindered progress.

### Views on the ECAU Website

All ECAU participants found the psychoeducation “not boring” but also too brief, although 1 respondent considered the succinctness of the information as something positive.

## Discussion

### Principal Findings

This study aimed to evaluate the feasibility of the STARS intervention and study among young people in Jordan. Key findings include that the trial procedures were adequate and overall views of STARS were positive, with some suggested improvements. As this was a feasibility study, the sample size was not powered to find a significant effect. Nonetheless, we did not find any differences between conditions over time that were statistically significant.

This study showed that trial procedures were feasible in terms of recruitment, dropout rates from the intervention, and retention at postassessment. The target sample size of 60 participants was reached within 2 months, mostly through word-of-mouth, with limited investment in social media advertisements, suggesting the suitability of online recruitment for a fully powered RCT. In terms of the recruited population, the majority reported a higher level of education and a Jordanian background. The internal consistency of the online-administered measures was good, and the item nonresponse was low [44], indicating that the set of questionnaires selected was acceptable. Adherence to the STARS chatbot lessons was adequate, with 73% of participants starting the intervention and 37% completing at least 8 chatbot lessons. These findings are in line with meta-analytic evidence on adherence rates in smartphone-delivered interventions for mental health problems [45] and comparable to what has been found for the Step-by-Step intervention studies in Lebanon, where 19% [14] and 32% [13] completed the intervention. Furthermore, retention at postassessment was good (80%) compared with what is commonly observed in smartphone-delivered interventions [13,14,46]. During the trial, no serious adverse events were reported.

Stakeholder views on the STARS intervention—identified through qualitative interviews—were overall positive. Participants generally liked the chatbot interface and found the therapeutic skills useful. Several aspects hampered easy use of the chatbot, including technical barriers such as bugs and internet connection problems that prevented continuing lessons; access barriers such as reliance on email for login; and restrictions to the intervention’s flexibility such as content spacing, predefined response options, and limited availability of e-helpers. Improvements to the chatbot within the confines of the system will be made prior to the main RCT. For example, it is not feasible to use mainly open-text responses as it does not use machine learning.

Adherence to the e-helper support calls was relatively low, with only 13% of participants attending all 5 e-helper support calls. In addition, although the fidelity review of e-helper support calls indicated that most aspects of the calls were carried out, the calls were short in duration. The process evaluation interviews also revealed that some participants felt e-helpers could be more caring, and e-helpers expressed a need for more guidance on how to carry out the calls more effectively. These very important findings together point to a need for increased support for e-helpers, for example, more detailed call scripts that address common issues and additional training and supervision. This will be addressed for the main RCT.

Limitations of this study include the use of only an immediate postassessment, while the primary end point of the fully powered RCT will be at a 3-month follow-up. This study thus does not provide an estimate of the retention rate at the 3-month follow-up. Second, recruitment of participants was mainly done through IFH peer educators and universities and limited posts on social media, while for a fully powered RCT, the focus will likely be more on large social media advertisements. Finally, this study did not include an active control group, which did not allow delineation of the STARS content from nonspecific effects of engaging with a digital intervention. STARS needs to be initially evaluated to determine its efficacy, but subsequent dismantling studies are needed to determine the active components of the program by comparing STARS against more active controls. Strengths include the use of both quantitative and qualitative research methods allowing for identification of more subtle use and engagement factors with both the chatbot and e-helper calls that would not have been possible with quantitative data alone. This study aimed to test the feasibility of STARS prior to a fully powered definitive RCT to evaluate the effectiveness of STARS. We expect the fully powered RCT to provide an important contribution to the field in terms of the effectiveness of delivering such digital mental health interventions in countries with limited mental health resources, particularly as most mental health chatbots have been evaluated in higher income country settings [47].

### Conclusion

This feasibility RCT showed that the newly developed digital STARS intervention is feasible for use by young people in Jordan. The study revealed several aspects of the STARS intervention that could be improved, especially aspects related to ease of accessing the chatbot, flexibility in its use, and e-helper support.

## Data Availability

Data will be available from the corresponding author upon reasonable request.

## Appendix

Multimedia Appendix 1 CONSORT checklist.

## Abbreviations

AI: artificial intelligence
ANCOVA: analysis of covariance
CONSORT: Consolidated Standards of Reporting Trials
CSQ-I: Client Satisfaction Questionnaire for internet-based interventions
ECAU: enhanced care as usual
HSCL-25: Hopkins Symptom Checklist-25
IFH: Institute of Family Health
K10: Kessler Psychological Distress Scale
LMIC: low- and middle-income countries
PSYCHLOPS: Psychological Outcomes Profiles
RCT: randomized controlled trial
STARS: Scalable Technology for Adolescents and youth to Reduce Stress
WHO: World Health Organization
WHO-5: WHO-Five Well-Being Index
WHODAS: WHO Disability Assessment Schedule
